# Comparison of Nevirapine Plasma Concentrations between Lead-In and Steady-State Periods in Chinese HIV-Infected Patients

**DOI:** 10.1371/journal.pone.0052950

**Published:** 2013-01-24

**Authors:** Huijuan Kou, Xiaoli Du, Yanling Li, Jing Xie, Zhifeng Qiu, Min Ye, Qiang Fu, Yang Han, Zhu Zhu, Taisheng Li

**Affiliations:** 1 Department of Infectious Diseases, Peking Union Medical College Hospital, Chinese Academy of Medical Sciences, Beijing, China; 2 Department of Gerontology, Sichuan Provincial People's Hospital, Sichuan Academy of Medical Sciences, Chengdu, China; 3 Department of Pharmacy, Peking Union Medical College Hospital, Chinese Academy of Medical Sciences, Beijing, China; National AIDS Research Institute, India

## Abstract

**Objectives:**

To investigate the potential of nevirapine 200 mg once-daily regimen and evaluate the influence of patient characteristics on nevirapine concentrations.

**Methods:**

This was a prospective, multicentre cohort study with 532 HIV-infected patients receiving nevirapine as a part of their initial antiretroviral therapy. Plasma samples were collected at trough or peak time at the end of week 2 (lead-in period) and week 4, 12, 24, 36, and 48 (steady-state period), and nevirapine concentrations were determined using a validated HPLC method. Potential influencing factors associated with nevirapine concentrations were evaluated using univariate and multivariate logistic regression.

**Results:**

A total of 2348 nevirapine plasma concentrations were collected, including 1510 trough and 838 peak values. The median nevirapine trough and peak concentration during the lead-in period were 4.26 µg/mL (IQR 3.05–5.61) and 5.07 µg/mL (IQR 3.92–6.44) respectively, which both exceeded the recommended thresholds of nevirapine plasma concentrations. Baseline hepatic function had a moderate effect on median nevirapine trough concentrations at week 2 (4.25 µg/mL *v.s.* 4.86 µg/mL, for ALT <1.5×ULN and ≥1.5×ULN, respectively, *P* = 0.045). No significant difference was observed in median nevirapine trough concentration between lead-in and steady-state periods in patients with baseline ALT and AST level ≥1.5×ULN (*P* = 0.171, *P* = 0.769), which was different from the patients with ALT/AST level <1.5ULN. The median trough concentrations were significantly higher in HIV/HCV co-infected patients than those without HCV at week 48 (8.16 µg/mL *v.s.* 6.15 µg/mL, *P* = 0.004).

**Conclusions:**

The 200 mg once-daily regimen of nevirapine might be comparable to twice-daily in plasma pharmacokinetics in Chinese population. Hepatic function prior to nevirapine treatment and HIV/HCV coinfection were significantly associated with nevirapine concentrations.

**Registration:**

Clinicaltrial.gov ID: NCT00872417

## Introduction

Nevirapine is a human immunodeficiency virus type 1 (HIV-1) specific non-nucleoside reverse transcriptase inhibitor that binds directly to the viral reverse transcriptase of HIV-1 to block polymerase activity by causing disruption of the enzymes catalytic site [1]. Combination antiretroviral therapy (cART) with nevirapine has been proven safe and effective in HIV-infected individuals. As a result, nevirapine is frequently used as a part of first-line regimens for the management of treatment-naive patients in resource-limited countries. It is typically dosed at 200 mg once daily during the first 2 weeks (lead-in period) and 200 mg twice daily thereafter (steady-state period), due to metabolic auto-induction of cytochrome P450 isoenzymes [1].

The substantial benefits conferred by cART, however, require strict patient adherence to the prescribed medication because poor adherence will lead to virologic failure. Adherence might be improved with more convenient dosing regimen. A possible measure for simplifying antiretroviral therapy is the use of once-daily dosing regimen. The long plasma elimination half-life of nevirapine (∼25 to 30 h) after multiple dosing may justify once daily dosing throughout the therapy with equivalent efficacy to that of twice daily regimen [2]–[4]. However, several clinical trials [3], [5] investigating 400 mg once-daily dosing resulted with significantly lower trough (*C*_trough_) and higher peak concentrations (*C*_max_). Because high nevirapine concentrations are associated with increased adverse events [6]–[12] and low plasma concentration may lead to virologic failure and drug resistance [13], [14], adoption of once-daily dosing regimen in clinical practice remains controversial.

Our group has found that the pharmacokinetic profiles of nevirapine in Chinese patients are different with those in Caucasians and blacks [15]. It was demonstrated that patients treated with standard nevirapine dosing regimen, *i.e.* 200 mg twice daily, had excessively high plasma concentrations, which may contribute to the higher prevalence of hepatotoxicity in these patients. Another prospective study [16] further confirmed a significant positive correlation between nevirapine *C*_trough_ and liver toxicity among Chinese HIV-infected patients, especially in males. To date, Food and Drug Administration approves one-pill, once-daily nevirapine extended-release tablets for use in combination with other antiretroviral agents for treatment of HIV-1 infection in adults. In the context of attempts to simplify treatment regimen while securing efficacy, reducing toxicity and enhancing adherence, there is great interest in nevirapine 200 mg once daily dosing for the treatment of Chinese HIV-infected patients in a long run. The present study aims to investigate the potential of nevirapine 200 mg once daily regimen in Chinese HIV-infected patients by comparing nevirapine plasma concentrations during the lead-in period with the steady-state concentrations. The influence of patient characteristics was evaluated on nevirapine concentrations.

## Results

### Baseline characteristics

**Figure 1:**
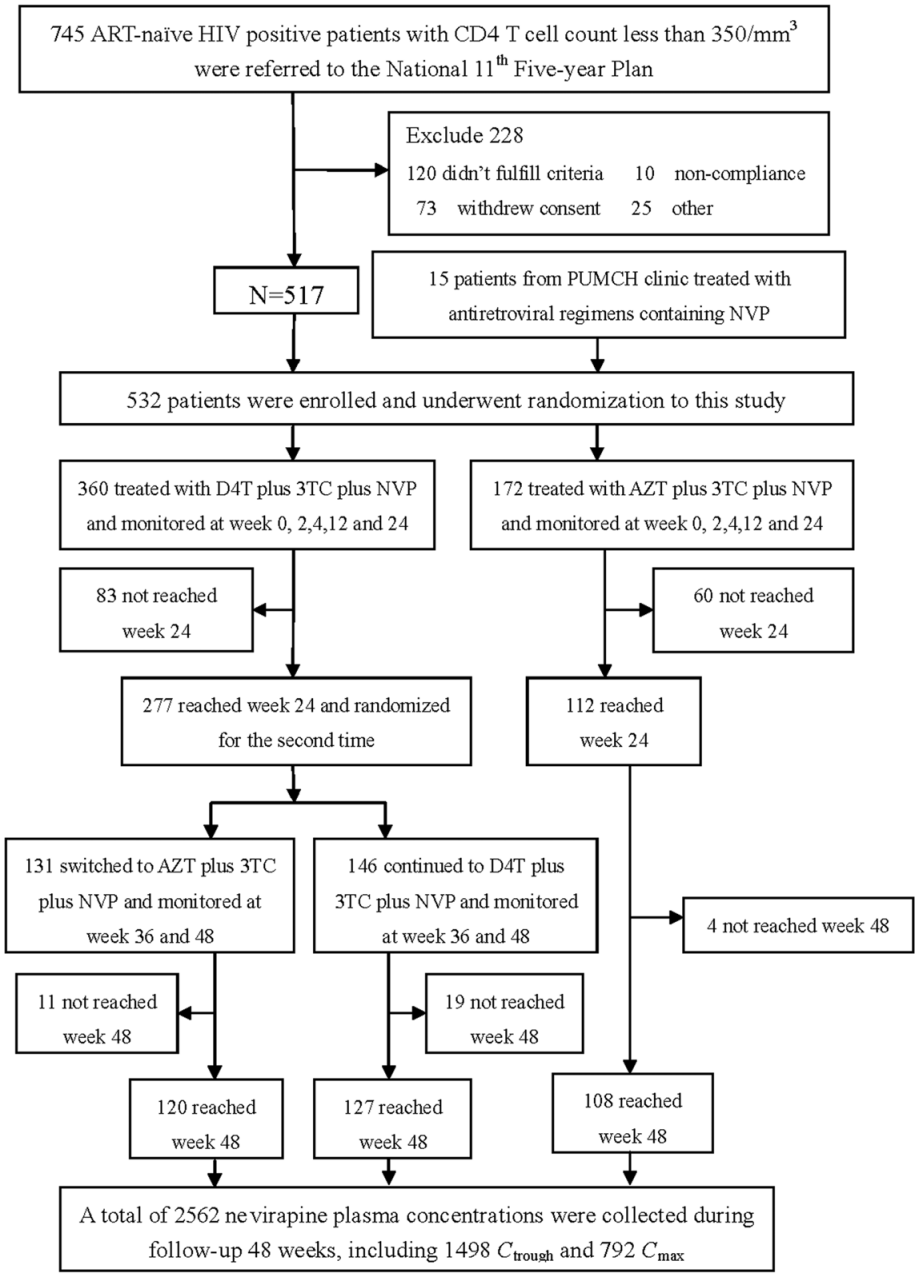
Flow chart of participants through the clinical trial. ART: antiretroviral therapy; AZT: zidovudine; NVP: nevirapine; 3TC: lamivudine; D4T: stavudine; C_trough_: trough concentrations of nevirapine; C_max_: peak concentrations of nevirapine.

Five hundred and thirty-two (n = 532) eligible HIV-infected patients were included and completed this study (Figure 1). Demographic characteristics of these patients were summarized in Table 1. There were 265 males and 95 females in d4T group and 132 males and 40 females in AZT group. Forty-three patients with ALT level ≥1.5×ULN and 23 with AST level ≥1.5×ULN at baseline were enrolled. Seventy-four (14%) patients were HBV co-infected and 64 (12%) were HCV co-infected.

**Table 1:** Baseline demographic characteristics of patients.

	D4T+3TC+NVP (n = 360)	AZT+3TC+NVP (n = 172)	All patients (n = 532)
**Gender - n (%)**			
Male	265 (73.61)	132 (76.74)	397 (74.62)
Female	95 (26.39)	40 (23.26)	135 (25.38)
**Age (yrs) - median (IQR)**	37 (30, 46)	37 (30, 44)	37 (30, 45)
**Duration of illness (months) - median (IQR)**	21.3(18.27,29.66)	21.3(18.27,31.43)	21.3(18.27,30.43)
**Route of transmission - n (%)**			
Homosexual	136 (37.78)	72 (41.86)	208 (39.10)
Heterosexual	150 (41.67)	71 (41.28)	221 (41.54)
Blood	36 (10.00)	15 (8.72)	51 (9.59)
Others	38 (10.56)	14 (8.14)	52 (9.77)
**Body mass index (Kg/m^2^) - mean±SD**	21.57±3.01	20.97±2.60	21.37±2.89
**ALT level (U/L) - median (IQR)**	24 (17, 35)	22.5 (16, 40)	23 (17, 36)
**AST level (U/L) - median (IQR)**	25 (21, 33)	25 (21, 35)	25 (21, 34)
**Hepatitis B coinfection -n (%)**			
positive	50 (13.89)	24 (13.95)	74 (13.91)
negative	303 (84.16)	145 (84.30)	448 (84.21)
unknown	7(1.94)	3(1.74)	10(1.88)
**Hepatitis C coinfection -n (%)**			
positive	47 (13.06)	17 (9.88)	64 (12.03)
negative	305 (84.72)	149 (86.63)	454 (85.34)
unknown - n (%)	8(2.22)	6(3.49)	14(2.63)
**CD4 cell counts (cells/mm^3^) - mean±SD**	172±98	174±105	173±100
**HIV-1 RNA level (log10 copies) - mean±SD**	4.52±0.74	4.56±0.78	4.53±0.75

ALT: alanine aminotransferase.

AST: aspartate aminotransferase.

ULN: upper limits of normal.

The presence of hepatitis B (HBV) or hepatitis C (HCV) was determined by the identified of HBV surface antigen or HCV antibodies.

### Comparison of nevirapine trough concentrations

**Figure 2:**
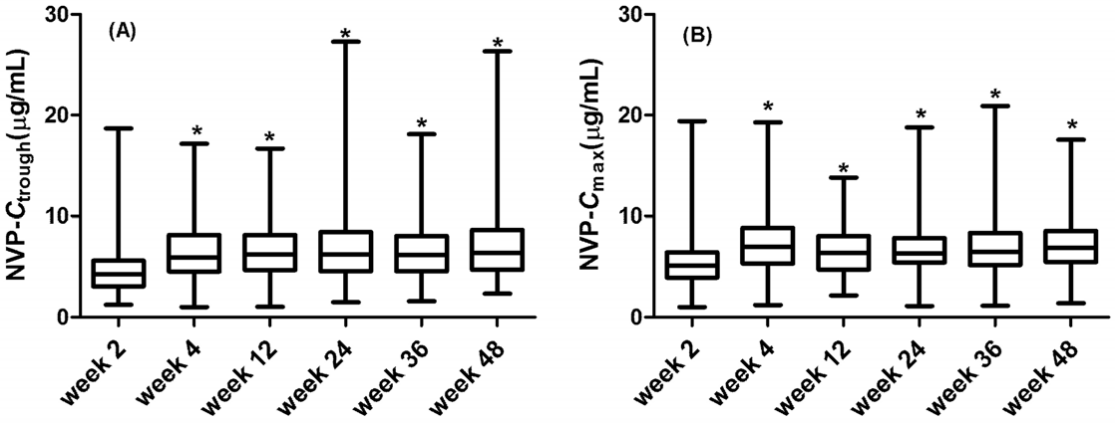
Comparison of nevirapine plasma concentrations between lead-in and steady-state periods during follow-up 48 weeks. The differences between lead-in and steady-state periods for nevirapine (A) trough (*P*<0.0001) and (B) peak (*P*<0.0001) concentrations were assessed by the Kruskal-Wallis H test.

A total of 1510 nevirapine *C*_trough_ were available during the 48-week period, of which 290 at week 2, 270 at week 4, 247 at week 12, 244 at week 24, 233 at week 36 and 226 at week 48. Figure 2 (A) shows that the median *C*_trough_ was 4.26 µg/mL (IQR 3.05–5.61) at the end of week 2 when the patients were administered with nevirapine 200 mg once daily, significantly lower than those in the later weeks when patients were receiving twice-daily dosing (6.15 µg/mL, IQR 4.63–8.20) (*P*<0.0001). The steady-state nevirapine *C*_trough_ at week 4, 12, 24, 36 and 48 were 5.90 µg/mL (IQR 4.54–8.14), 6.21 µg/mL (IQR 4.68–8.15), 6.23 µg/mL (IQR 4.56–8.47), 6.15 µg/mL (IQR 4.51–8.07) and 6.37 µg/mL (IQR 4.72–8.64), respectively (Table 2).

**Table 2:** Nevirapine plasma concentrations during follow-up 48 weeks.

	2W	4W	12W	24W	36W	48W
**n**	290	270	247	244	233	226
**C_trough_ (µg/mL)**	4.26 (IQR 3.05–5.61)	5.90 (IQR 4.54–8.14)	6.21 (IQR 4.68–8.15)	6.23 (IQR 4.56–8.47)	6.15 (IQR 4.51–8.07)	6.37 (IQR 4.72–8.64)
**n**	167	159	137	129	126	120
**C_max_ (µg/mL)**	5.07(IQR 3.92–6.44)	6.89 (IQR 5.23–8.84)	6.34 (IQR 4.72–8.03)	6.32 (IQR 5.40–7.85)	6.48 (IQR 5.19–8.35)	6.91 (IQR 5.49–8.56)

C_trough_: trough concentration of nevirapine.

C_max:_ peak concentration of nevirapine.

Data were described as median (IQR: inter-quartile range).

**Figure 3:**
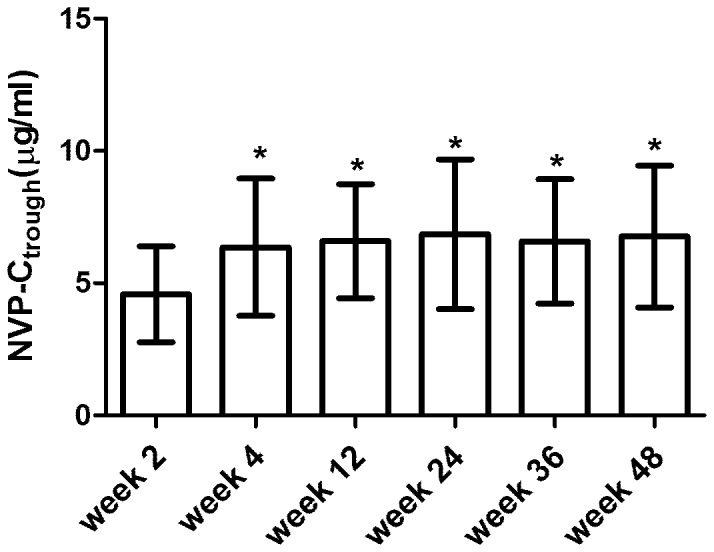
Comparison of nevirapine trough concentration in 105 patients with HIV infection. Analysis of variance was utilized to evaluate nevirapine trough concentration in 105 HIV+ patients with 6 consecutive points during follow-up 48 weeks (*P*<0.05).

One hundred and five HIV-infected adults (n = 105) had 6 consecutive *C*_trough_ during the follow-up period. A one-way analysis of variance (ANOVA) shows that the mean nevirapine *C*_trough_ (±SD) at the end of week 2 (4.59±1.81 µg/mL) was significantly lower than the levels at week 4 (6.40±2.59 µg/mL), week 12 (6.59±2.16 µg/mL), week 24 (6.85±2.83 µg/mL), week 36 (6.58±2.35 µg/mL), and week 48 (6.76±2.68 µg/mL) (Figure 3).

### Comparison of nevirapine peak concentrations

Altogether 838 *C*_max_ of nevirapine were obtained, of which 167 at week 2, 159 at week 4, 137 at week 12, 129 at week 24, 126 at week 36 and 120 at week 48. It was demonstrated in Figure 2 (B) that the median *C*_max_ of 5.07 µg/mL (IQR 3.92–6.44) during the lead-in period was significantly lower than the steady-state levels (6.51 µg/mL, IQR 5.22–8.41) (*P*<0.0001). The observed *C*_max_ of nevirapine at week 4, 12, 24, 36 and 48 were 6.89 µg/mL (IQR 5.23–8.84), 6.34 µg/mL (IQR 4.72–8.03), 6.32 µg/mL (IQR 5.40–7.85), 6.48 µg/mL (IQR 5.19–8.35) and 6.91 µg/mL (IQR 5.49–8.56), respectively (Table 2).

### Influence of patient characteristics on nevirapine concentrations

**Figure 4:**
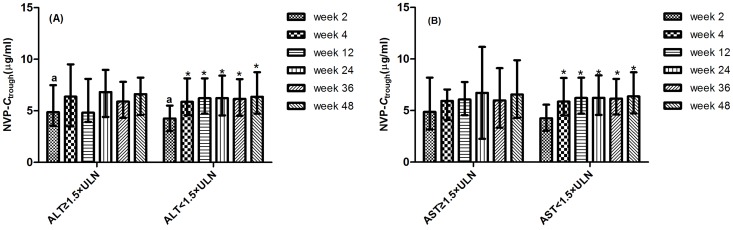
Relationship between nevirapine trough concentration and baseline liver function. The nevirapine trough concentration at week 2 in patients with baseline ALT level <1.5×ULN was significant lower than those with ALT ≥1.5×ULN at baseline (*P* = 0.045). No significant difference was observed between lead-in and steady-state periods in patients with (A) ALT level ≥1.5×ULN (*P* = 0.171) and (B) AST level ≥1.5×ULN (*P* = 0.769) at baseline.

In the univariate stratification analysis, the median *C*_trough_ of nevirapine at week 2 in patients with baseline ALT level <1.5×ULN was significant lower than those with ALT level ≥1.5×ULN at baseline (4.25 *v.s.* 4.86 µg/mL, *P* = 0.045) (Figure 4A). There was no significant association between *C*_trough_ at week 2 and gender, weight, HBV or HCV coinfection, baseline AST level, CD4 cell counts and viral load.

No significant difference was observed in median *C*_trough_ of nevirapine between lead-in and steady-state periods in patients with baseline ALT level ≥1.5×ULN (4.86 *v.s.* 6.12 µg/mL, *P* = 0.171) and baseline AST level ≥1.5×ULN (4.86 *v.s.* 5.94 µg/mL, *P* = 0.769), whereas the median *C*_trough_ during lead-in period was significantly lower than the steady-state levels in patients with ALT/AST <1.5 ULN prior to initial treatment (Figure 4).

**Figure 5:**
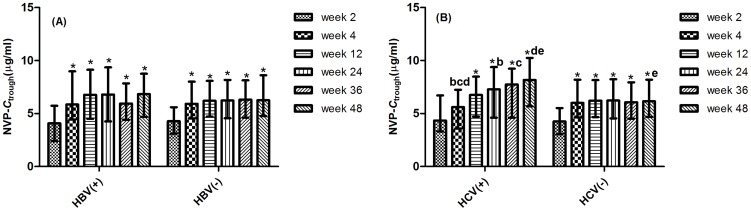
Relationship between nevirapine trough concentration and underlying liver diseases. The differences for nevirapine trough concentration between lead-in and steady-state periods was observed in patients with HBV infection (A). The nevirapine trough concentration at week 4 was significantly lower than the values at week 24, 36 and 48 (*P*<0.05) in patients with HCV infection (B). The nevirapine trough concentration at week 48 in patients with HCV infection was significant higher than those without HCV coinfection (*P* = 0.004).

Of the HIV and HCV co-infected patients, the median *C*_trough_ of nevirapine at week 4 was significantly lower than the values at week 24, 36 and 48 (5.60 *v.s.* 7.28, 7.75, 8.16 µg/mL, respectively, *P*<0.05). The median *C*_trough_ of nevirapine at week 48 in patients with HCV coinfection was significant higher than those without HCV coinfection (8.16 *v.s.* 6.15 µg/mL, *P* = 0.004). The difference was not observed in the patients with HBV coinfection (Figure 5).

In the univariate and multivariate logistic regression model, gender, age, weight, HBV or HCV coinfection, baseline dosing regimen, ALT or AST level, CD4 cell counts and viral load appeared to have no significant association with nevirapine trough concentration <3.0 µg/mL and <3.9 µg/mL at week 2, respectively (Table 3).

**Table 3:** Univariate and multivariate logistic model to examine risk factors to predict nevirapine trough concentration lower than recommended thresholds at week 2.

	Univariate 1			Univariate 2			Multivariate 1			Multivariate 2		
	OR	95%CI	*P* value	OR	95%CI	*P* value	OR	95%CI	*P* value	OR	95%CI	*P* value
**male**	1.569	0.840–2.928	0.155	1.132	0.645–1.987	0.666	0.576	0.280–1.183	0.133	0.967	0.512–1.827	0.919
**Age ≥50**	1.668	0.706–3.943	0.240	1.128	0.582–2.186	0.721	0.992	0.963–1.022	0.598	1.009	0.984–1.034	0.488
**Weight ≥50 Kg**	0.512	0.215–1.217	0.124	0.774	0.340–1.760	0.540	1.004	0.971–1.037	0.833	0.984	0.956–1.012	0.262
**Baseline ALT level ≥1.5×ULN**	1.830	0.610–5.491	0.275	1.813	0.766–4.292	0.171	1.002	0.980–1.024	0.867	1.005	0.987–1.023	0.581
**Baseline AST level ≥1.5×ULN**	0.973	0.306–3.093	0.964	1.035	0.382–2.801	0.946	0.996	0.965–1.028	0.821	0.990	0.965–1.016	0.465
**Baseline CD4 counts ≥250 cells/µL**	0.706	0.387–1.287	0.254	0.878	0.515–1.495	0.631	1.001	0.998–1.004	0.601	1.000	0.998–1.003	0.809
**Baseline viral load ≥5 log10 copies**	0.847	0.458–1.567	0.597	0.894	0.524–1.525	0.681	1.344	0.888–2.035	0.163	1.137	0.812–1.593	0.455
**HBV co-infection**	0.554	0.260–1.179	0.122	0.776	0.385–1.564	0.477						
**HCV co-infection**	1.241	0.542–2.842	0.610	0.965	0.491–1.898	0.918						

**Univariate 1 and multivariate 1:** The recommended cut-off value was 3.0 µg/mL.

**Univariate 2 and multivariate 2:** The recommended cut-off value was 3.9 µg/mL.

ALT: alanine aminotransferase; AST: aspartate aminotransferase.

## Discussion

In the present study, we compared the nevirapine plasma concentrations between lead-in and steady-state periods. The median *C*_trough_ and *C*_max_ of nevirapine during the lead-in period were 4.26 µg/mL and 5.07 µg/mL respectively, which both exceeded the current recommended thresholds of nevirapine plasma concentrations, *i.e.* 3.0 µg/mL [17] and 3.9 µg/mL [16]. From this perspective, the 200 mg once-daily dosing regimen of nevirapine is worth of further evaluation for its role in Chinese population. In addition, hepatic function prior to nevirapine treatment and HIV/HCV coinfection were significantly associated with nevirapine plasma concentrations.

The efficacy and safety of nevirapine 400 mg once daily in treatment of HIV-infected patients had been assessed in several studies [4], [18], [19]. No significant difference was shown between 400 mg once-daily and 200 mg twice-daily dosing. However, *van Heeswijk et al* [5] reported that *C*_min_ and *C*_max_ for nevirapine 400 mg once-daily regimen were significantly lower (2.88 *versus* 3.73 µg/mL) and higher (6.69 *versus* 5.74 µg/mL) compared with the 200 mg twice daily. The 2NN sub-study confirmed this findings and showed that *C*_min_ (3.26 *versus* 4.44 µg/mL) was lower and *C*_max_ (7.88 *versus* 6.55 µg/mL) was higher in the 400 mg once-daily dosing [3]. The 2NN study also demonstrated that high *C*_max_ might result in a higher incidence of toxicity in patients with nevirapine once-daily dosing than those assigned twice-daily administration [20]. The increased drug related adverse events, especially liver toxicity, remain significant obstacles to routine use of nevirapine 400 mg once daily dosing strategy.

The liver toxicity is one of the most common hypersensitivity reactions to nevirapine and may be associated with its plasma concentrations. *Gonzalez de Requena et al.* [8] investigated the effect of nevirapine plasma exposure on liver enzyme elevations and observed that among patients with chronic HCV coinfection, nevirapine concentrations >6 µg/mL were associated with a 92% risk of liver toxicity. Our previous studies [16], [21], [22] reported that a high frequency of liver toxicity was observed in Chinese HIV-infected patients when administered nevirapine twice-daily standard dosing, including approximate 23% patients with severe liver toxicity within 12 weeks of initial therapy. Most importantly, *Wang J et al.* [16] found a significant positive association between nevirapine *C*_trough_ and liver toxicity among Chinese HIV-infected patients, especially in males (*P* = 0.015).

The steady-state pharmacokinetic study in 15 Chinese patients with HIV infection [15] confirmed that the standard therapeutic regimens of nevirapine 200 mg twice daily led to longer half-life, higher concentrations and lower clearance of nevirapine than the values in Caucasians [5]. A non-compartment model was used to describe the pharmacokinetic parameters of nevirapine as median including t_1/2_ (30.94 h), AUC_0–12 h_ (92.82 µg·h/mL), Cl/F (0.71 L/h), *C*_max_ (10.09 µg/mL) and *C*_trough_ (7.88 µg/mL), respectively [15]. The mean *C*_trough_ in Chinese males and females (8.95 µg/mL and 6.59 µg/mL) were significantly higher than the levels in Caucasians and blacks [23] (3.34 µg/mL and 3.46 µg/mL). These findings indicated that nevirapine 200 mg twice daily dosing produced excessive drug load in plasma and might contribute to the higher prevalence of hepatotoxicity in Chinese patients. Considering the unfavorable safety associated with 400 mg once-daily in the literature, a lower dosage, nevirapine 200 mg once-daily dosing regimen, would be worthy of evaluation for clinical application.

The present study showed that the median *C*_trough_ of nevirapine 200 mg once daily at the end of week 2 was significantly lower than the twice-daily dosing in later weeks (4.26 versus 6.15 µg/mL, *P* = 0.000). Similarly, the median *C*_max_ of nevirapine during the lead-in period was also lower than the steady-state levels (5.07 versus 6.51 µg/mL, *P* = 0.000). The decreased nevirapine concentrations with 200 mg once daily dosing may suggest a lower incidence of liver toxicity than twice daily. To test this assumption, an small trial in seven treatment-naïve Chinese HIV-infected patients was carried out by our team. The seven HIV+ adults patients were administered with nevirapine 200 mg once daily as a part of initiating antiretroviral therapy. Till now, they maintained a high level of efficacy and comparable tolerability during 2-month follow up compared with the twice-daily dosing regimen.

On the other hand, there are some concerns about virologic failure which was related to low nevirapine plasma levels in clinical practice. The relationship between nevirapine concentration and virologic response has been explored in previous studies. The INCAS trial [24] suggested that a nevirapine plasma concentration range of 3.45–3.88 mg/ml at week 12 was predictive of virologic success after 52 weeks of therapy. *Vries-Sluijs et al.* [13] demonstrated that nevirapine plasma concentration ≤3.0 µg/mL was directly associated with risk of treatment failure. The guidelines from Department of Health and Human Services in the United States proposed a minimum target nevirapine *C*_trough_ of 3.0 µg/mL [17]. Our previous study [16] suggested a target cut-off value of nevirapine *C*_trough_ at 3.9 µg/mL for Chinese patients with HIV infection, higher than the commonly recommended 3.0 µg/mL. The current study demonstrated that median *C*_trough_ (4.26 µg/mL, IQR 3.05–5.61) and *C*_max_ (5.07 µg/mL, IQR 3.92–6.44) of nevirapine in Chinese HIV-infected patients receiving 200 mg once daily were above the recommended thresholds of nevirapine concentrations, suggesting that nevirapine 200 mg once daily regimen may produce adequate viral inhibition in Chinese HIV-infected patients. The ongoing pilot trial in Chinese patients confirmed this assumption. Certainly, we cannot rule out the possibility that some patients will have nevirapine levels lower than the target thresholds due to inter-individual variability and unpredictable features, which may result in virologic treatment failure and even drug resistance. So routine therapeutic drug monitoring should be carried out.

Previous studies have found ethnicity, gender, weight and underlying hepatic disease to be predictive of nevirapine plasma concentrations [25]–[32]. Our data confirmed that hepatic function prior to antiretroviral treatment was significantly associated with the nevirapine lead-in trough concentrations (4.86 µg/mL *v.s.* 4.25 µg/mL, for ALT level≥*v.s.*<1.5×ULN, *P* = 0.045) and might exert an influence on the metabolism and clearance of nevirapine at steady state, because no significant difference was observed in median *C*_trough_ of nevirapine between lead-in and steady-state periods in patients with baseline ALT and AST level ≥1.5×ULN (*P* = 0.171, *P* = 0.769), which was different from the patients with ALT/AST level <1.5×ULN. Gender, weight, HBV or HCV coinfection, baseline CD4 cell counts and viral load appeared to have no significant influence on nevirapine plasma concentrations. Consistently, the univariate and multivariate logistic regression models showed that none of the examined factors was found to predict nevirapine trough concentration at week 2 lower than 3.0 µg/mL or 3.9 µg/mL.

The nevirapine trough concentration increased gradually in HIV/HCV coinfected patients during the follow-up periods and finally was significantly higher than those patients without HCV coinfection at week 48 (8.16 *v.s.* 6.15 µg/mL, *P* = 0.004). We assumed that a high incidence of liver toxicity in Chinese patients and particularly a great risk of severe hepatotoxicity in HIV/HCV coinfected patients were both significantly associated with plasma nevirapine exposure. This finding was consistent with our previous study [16] and further confirmed that nevirapine 200 mg twice daily dosing produced excessive drug load in plasma and dosage adjustment based on therapeutic drug monitoring would be necessity in Chinese HIV-infected patients, especially in those with HCV co-infected.

To date, an increasing evidence demonstrated that host genetic polymorphisms may in part explain the observed inter-individual variability of drug disposition and response [29]–[32]. In an ethnically diverse population, both non-Caucasian ethnicity and carriage of the variant allele of CYP2B6 G516T single nucleotide polymorphism, which linked to the altered enzyme function, were significant predictors of nevirapine C_trough_. It was indicated that Chinese patients with CYP 2B6 G516T polymorphism reduced enzyme function leading to a greater plasma exposure of nevirapine. The impact of weight should also be considered when explaining the difference in nevirapine drug concentration. The previous study found that higher body weight was significantly associated with lower nevirapine concentration [27], it was indicated that Chinese patients with relatively lower body weight were more likely to achieve an adequate drug level with once-daily nevirapine dosing compared with the Caucasians.

Several limitations of our study must be addressed. Firstly, due to inter-patient variability of nevirapine *C*_max_, *i.e.* the exact peak time for nevirapine is different from patient to patient, influencing factors affected *C*_max_ was not evaluated using univariate and multivariate analyses. Secondly, unrecognized confounders may have affected nevirapine concentrations, *i.e.* dosing in relation to food, concurrent medications, or genetic polymorphisms of CYP2B6, which may significantly influenced nevirapine metabolism and clearance. Lastly, all these patients were administered nevirapine according to the international treatment guidelines, *i.e.* 200 mg once daily for 14 days followed by 200 mg twice daily. So the plasma nevirapine concentration for 200 mg once daily could only be obtained for as long as 2 weeks. It was impossible to get the long-term efficacy and safety data of this dosing regimen. Although the pilot clinical trial mention above suggested that this regimen was safe and effective during 8-week period, however, the cohort was small and the follow-up was short. A large and long-term prospective clinical study is necessary to fully evaluate the efficacy and safety of this regimen.

In conclusion, this is the first report demonstrating that the 200 mg once daily dosing regimen might produce adequate plasma nevirapine concentrations for both inhibiting HIV and reducing hepatic toxicity in Chinese population, which is worth of further evaluation in a prospective randomized study. Hepatic function prior to antiretroviral treatment and HIV/HCV coinfection were found to be significantly associated with the nevirapine concentrations. The benefit of dosage adjustment based on therapeutic drug monitoring among Chinese HIV-infected patients would optimize nevirapine containing antiretroviral therapy.

## Materials and Methods

### Patients

A prospective, randomized and multicenter cohort study was conducted in 10 clinical units located in China, including Peking Union Medical College Hospital, Beijing Youan Hospital, Beijing Ditan Hospital, Zhengzhou Infectious Diseases Hospital, Xi'an Tangdu Hospital, Shanghai Public Health Center, Shenzhen CDC, The 8^th^ Hospital of Guangzhou, Fuzhou Infectious Diseases Hospital and HIV/AIDS Care Center of Yunnan.

Patients were recruited from January 2009 to December 2010. Male and female antiretroviral-naïve patients with documented HIV-1 infection were eligible for inclusion if they were between the age of 18 and 65 years with CD4+T cell count <350 cells/mm^3^ for more than 4 weeks. Main exclusion criteria were acute HIV infection, AIDS-defining illness within 2 weeks of entry, alcohol and injection drug users, acute or chronic pancreatitis, severe peptic ulcers, severe psychiatric and neurologic diseases; if female, pregnant, breastfeeding, or of child-bearing potential and not using adequate contraception. Laboratory exclusion criteria included white blood cell <2.0×10^9^/L, absolute neutrophil count <1.0×10^9^/L, hemoglobin level <90 g/L, or platelet count <75×10^9^/L, transaminase and alkaline phosphatase level >3 times upper limits of normal value (×ULN) and serum creatinine level >1.5×ULN. Another important exclusion criterion was non-adherence to the study treatment regimen which was defined as less than 95% adherence.

### Study design

The cohort study was approved by institutional review boards and carried out in accordance with the Declaration of Helsinki and the principles of Good Clinical Practice. Written informed consent was obtained from each patient and the study protocol was approved by the ethics committee of Peking Union Medical College Hospital. The protocol for this trial and supporting CONSORT checklist are available as supporting information; see Checklist S1 and Protocol S1.

All the patients received a standard ART regimen based on nevirapine together with two nucleoside reverse transcriptase inhibitors including stavudine plus lamivudine or zidovudine plus lamivudine. Nevirapine (Desano Pharma, Shanghai, China) was administered at 200 mg once daily for 2 weeks and 200 mg twice daily thereafter. The bioequivalence of nevirapine in reference to Viramune® from Boehringer Ingelheim was demonstrated in the previous study [33].

During the treatment period, patients were monitored at baseline, week 2, 4, 12, 24, 36, and 48 for clinical features (particularly severe adverse events), plasma nevirapine concentrations and laboratory values including blood routine examination, hepatic and renal function, and hepatitis B (HBV) or hepatitis C (HCV) serological state.

### Sampling and bioanalysis

#### Sample collecting

Blood samples were drawn prior to the next drug administration for *C*_trough_ and/or 2 h post-ingestion for *C*_max_. All samples were collected in spray dry powdered EDTA tubes and centrifuged in the real time to obtain plasma, which were stored at −80°C and thawed at the day of analysis. The exact time of nevirapine dose and blood sampling was recorded.

#### Plasma nevirapine concentration determination

The nevirapine concentration in plasma was determined by a validated HPLC assay modified from a previous study [34]. The nevirapine concentration were analyzed on a Shim-pack CLC-ODS column (6 mmID×15 cm, 5 µm) with a mobile phase consisting of water-acetonitrile (23∶77) at a flow rate of 1 mL/min, and the wavelength for detection was 260 nm. Tegafur was used as an internal standard. The calibration curve of nevirapine was linear in the range of 0.05–10 µg/mL (r = 0.9999), and the limit of detection was 0.05 µg/mL. The RSDs of intra- and inter- run validations were less than 7%. The mean recoveries fell in the range of 90–110% for the high, middle and low concentrations. The nevirapine plasma samples demonstrated satisfactory stability.

#### Plasma HIV-1 viral load analysis

Plasma HIV-1 RNA viral load was measured by either bDNA Analyzer System 340 (Siemens, Germany) or the COBAS Ampliprep/TaqMan 48 (Roche, USA) according to the manufacturer's instructions. The measurement ranges were 50–500,000 copies/mL and 40–1,000,000 copies/mL, respectively.

#### CD4+T cell counts analysis

PBMCs were stained with combinations of immunofluorescent monoclonal antibodies FITC-CD3 and PEcy5-CD4 (Beckman-Coulter and Immunotech, USA) followed by flow cytometer analysis (3-color EPICS-XL flow cytometer, Beckman-Coulter Inc., USA) to determine the number of CD4+T cells (CD4+CD3+).

### Statistical analysis

Statistical analyses were performed with Statistical Product and Service Solutions for Windows (SPSS, version 13.0). Mean (± standard deviation, SD), median (interquartile range at 25th and 75th, IQR) and frequencies (%) were used to describe characteristics of patients as appropriate. Normal distribution of values was examined by Kolmogorov-Smirnov methods. Categorical variables were tested with Chi-Square or Fisher's exact test, and continuous variables were tested with Kruskal-Wallis or student's t test or one-way ANOVA.

The differences between lead-in and steady-state periods for nevirapine plasma concentrations were assessed by the Kruskal-Wallis H test. ANOVA was used to determine whether there were significant differences in nevirapine C_trough_ at the 6 follow-up visits in the 105 patients. An independent t-test or Mann-Whitney U test was used when two groups or two variables were compared.

Factors affecting nevirapine plasma exposure were estimated using univariate stratification analysis. Risk factors predicting nevirapine trough concentration at week 2 lower than the recommended cut-off thresholds were evaluated using univariate and multivariate logistic regression. Odd ratios (OR) and 95% confidence intervals (95% CI) were also obtained.

For all tests, P<0.05 was considered to be statistically significant.

## Supporting Information

Checklist S1 **CONSORT Checklist.**
(DOC)

Protocol S1 **Trial Protocol.**
(DOC)
