# Supplementary material for: Comparison of Nevirapine Plasma Concentrations between Lead-In and Steady-State Periods in Chinese HIV-Infected Patients
Source: PLoS One. 2013 Jan 24;8(1):e52950. doi: 10.1371/journal.pone.0052950 (PMC3554734; doi:10.1371/journal.pone.0052950)
Supplement: Protocol S1 — Trial Protocol. (DOC) [file pone.0052950.s002.doc]

**Trial Protocol**

**Study on the Antiviral Therapy and Immune Reconstitution of Chinese HIV/AIDS Patients**

The recruitment status of this study is unknown because the information has not been verified recently.

Verified March 2009 by Peking Union Medical College. Recruitment status was Not yet recruiting.

First Received on March 30, 2009. No Changes Posted

| Sponsor: | Peking Union Medical College |
| --- | --- |
| Collaborator: | Ministry of Science and Technology of the People´s Republic of China |
| Information provided by: | Peking Union Medical College |
| ClinicalTrials.gov Identifier: | NCT00872417 |


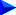
Purpose

This study will recruit 520 treatment-naive and 150 treatment-experienced patients to take the first line or second line of antiviral therapy. This study aims to set up a well-trained clinical and laboratory team in China, to explore the effects and side-effects of the first-line and the second line of ARV treatment in Chinese HIV/AIDS adult patients, to investigate the side-effects of ARV drugs, such as hepatotoxicity, lipoatrophy, cardiovascular influence, to explore the pharmacokinetics/pharmacodynamics (PK/PD) of Chinese generic ARV regiments and effective drug concentrations and to explore primary and secondary drug resistance in China and the immune reconstitution characters of long term ARV in Chinese adult AIDS patients. This study might provide more practical and optimizing prove for the treatment guideline for resource limited areas.

| [Condition](http://www.clinicaltrials.gov/ct2/help/conditions_desc) | [Intervention](http://www.clinicaltrials.gov/ct2/help/interventions_desc) | [Phase](http://www.clinicaltrials.gov/ct2/help/phase_desc) |
| --- | --- | --- |
| Acquired Immune Deficiency Syndrome HIV Infections | Drug: first line ARV (3TC+NVP+D4T or 3TC+NVP+AZT) Drug: second line ARV therapy (3TC+TDF+LPV/RTV) | Phase 4 |

| Study Type: | Interventional |
| --- | --- |
| Study Design: | Allocation: Randomized Endpoint Classification: Safety/Efficacy Study Intervention Model: Parallel Assignment Masking: Single Blind (Investigator) Primary Purpose: Treatment |
| Official Title: | Research on the Antiretroviral Therapy and Immune Reconstitution on Chinese HIV/AIDS Patients |

Resource links provided by NLM:

[Genetics Home Reference](http://ghr.nlm.nih.gov/) related topics: [complement factor I deficiency](http://www.clinicaltrials.gov/ct2/bye/AQoPWw4lZXcilwpxudhWudNzlXNiZip90dcx5Q1PedcOZBc9mwhazd7HuiYLNB7gWd-nmQDL5676eBczzdNgx.)

[MedlinePlus](http://www.nlm.nih.gov/medlineplus/) related topics: [HIV/AIDS](http://www.clinicaltrials.gov/ct2/bye/ZQoPWw4lZX-i-iSxudhWudNzlXNiZip9m67PvQ7xzwhaLwS9lQ16kQ7PFXNkWd7E.)

[Drug Information](http://druginfo.nlm.nih.gov/drugportal/drugportal.jsp) available for: [Stavudine](http://www.clinicaltrials.gov/ct2/bye/YQoPWw4lZXcPSi7iedN6ZXNxvdDxuQ7Ju6c9cXcPSi7iEd-yWB7EZ6o35Q1yzB-VuQUgEscxkd7898-PkiUH5Q7xz.) [Lamivudine](http://www.clinicaltrials.gov/ct2/bye/4QoPWw4lZXcPSi7iedN6ZXNxvdDxuQ7Ju6c9cXcPSi7iEd-yWB7EZ6o35Q1yzB-VuQUgEscxkd789ChGmQ16L6hzu6V.)

[U.S. FDA Resources](http://www.clinicaltrials.gov/ct2/info/fdalinks)

Further study details as provided by Peking Union Medical College:

Primary Outcome Measures:

- To set up a platform of antiviral therapy network all of CHINA, to obtain evidence to make first line or second line ARV treatment strategy for HIV/AIDS patients in resource limited areas. [ Time Frame: two years ] [ Designated as safety issue: Yes ]

Secondary Outcome Measures:

- Set up our own antiviral therapy guideline and drug side-effects, drug concentration and immune reconstitution result. [ Time Frame: two years ] [ Designated as safety issue: Yes ]

| Estimated Enrollment: | 750 |
| --- | --- |
| Study Start Date: | March 2009 |
| Estimated Study Completion Date: | December 2010 |
| Estimated Primary Completion Date: | December 2009 (Final data collection date for primary outcome measure) |

| [Arms](http://www.clinicaltrials.gov/ct2/help/arm_group_desc) | [Assigned Interventions](http://www.clinicaltrials.gov/ct2/help/interventions_desc) |
| --- | --- |
| Experimental: Treatment-naive  To explore the efficiency and safety of generic antiretroviral drugs for 520 treatment-naive HIV/AIDS patients | Drug: first line ARV (3TC+NVP+D4T or 3TC+NVP+AZT)  use the generic regimens: 3TC+NVP+D4T or 3TC+NVP+AZT to initiate the ARV therapy, after 6 months, half of the group 3TC+NVP+D4T patients switch to the the treatment of 3TC+NVP+AZT |
| No Intervention: TREATMENT-EXPERIENCED  To explore the long term ARV of treatment-experienced patients who have no sign of drug resistance; to explore the long term efficiency and safety and drug sife effects of ARV in HIV/AIDS patients. These patients have taken ARV for approximately 3 years already. |  |
| Experimental: drug resistance  To explore the second line drugs for those drug resistance patients | Drug: second line ARV therapy (3TC+TDF+LPV/RTV)  Use 3TC+TDF+LPV/RTV to treat those drug resistance patients, to explore the efficiency and safety of the second line ARV available in China |

Detailed Description:

Three arms will be studied in this research, 520 naive-treatment patients would be randomized to two groups, taking the generic drugs 3TC+D4T+NVP or AZT+3TC+NVP, 6 months later half of the 3TC+D4T+NVP group will switch to AZT+3TC+NVP, in order to observe the efficiency and safety of the first line drugs. Arm 2 will recruit 100 patients who are taking ARV for about three years already. Arm 3 will recruit 150 patients who have a Viral load of more than 1000 copies/ml, i.e., drug resistance. The second line drug 3TC+TDF+LPV/RTV will given to them and the safety and efficiency will be observed. All patients should be explored in terms of the clinical features, drugs side-effects, and immunological and viral response. The drug concentration and the metabolism changes would be explored also. Also the immune reconstitution will be studied for all patients. This study will be the first large-scale, multicentered, randomised, prospective ARV therapy study in China for HIV/AIDs patients. The result would provide proves for further practical antiviral therapy for China or other resource limited countries.


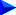
Eligibility

| Ages Eligible for Study: | 18 Years to 65 Years |
| --- | --- |
| Genders Eligible for Study: | Both |
| Accepts Healthy Volunteers: | No |

Criteria

Inclusion Criteria:

- age between 18-65 years
- HIV seropositive and confirmed by western blot
- antiretroviral therapy naive for arm 1
- CD4 cell count < 350/mm3
- good adherence and follow up in the same place

Exclusion Criteria:

- pregnancy and breastfeeding
- AIDS defining illness or any infectious disease occured in one month but still unstable within 14 days
- with WBC < 2000/ul, neutrophil count < 1000/ul, hemoglobin < 9 g/dl, platelet count < 75000/ul, amylase > 2 ULN, transaminase or alkaline phosphatase or total bilirubin > 2 ULN, creatinine > 2 ULT.
- present acute or chronic pancreatitis
- intravenous drug user
- peripheral nephropathy
- severe nephropathy or mental disorder
- severe gastral ulcer
- heart or brain arthrosclerosis


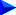
Contacts and Locations

Please refer to this study by its ClinicalTrials.gov identifier: NCT00872417
Contacts

| Contact: Tai sheng LI, M.D | 00861065295086 | [litsh@263.net](mailto:litsh@263.net?subject=NCT00872417, CACT0810, Study on the Antiviral Therapy and Immune Reconstitution of Chinese HIV/AIDS Patients) |
| --- | --- | --- |

Locations

| China | |
| --- | --- |
| Peking Union Medical College Hospital | Not yet recruiting |
| Beijing, China, 100730 | |
| Contact: Yang HAN [hanyang@pumch.cn](mailto:hanyang@pumch.cn?subject=NCT00872417, CACT0810, Study on the Antiviral Therapy and Immune Reconstitution of Chinese HIV/AIDS Patients) | |
| Sub-Investigator: Wei LU, M.D | |

Sponsors and Collaborators

Peking Union Medical College

Ministry of Science and Technology of the People´s Republic of China

Investigators

| Study Chair: | Tai sheng LI, M.D | PUMCH |
| --- | --- | --- |


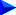
More Information

No publications provided

Additional publications automatically indexed to this study by ClinicalTrials.gov Identifier (NCT Number):

Wang J, Kou H, Fu Q, Han Y, Qiu Z, et al. (2011) Nevirapine Plasma Concentrations Are Associated with Virologic Response and Hepatotoxicity in Chinese Patients with HIV Infection. PLoS ONE 6(10): e26739. doi:10.1371/journal.pone.0026739

| Responsible Party: | Li Taisheng, MD, Peking Union Medical College Hospital |
| --- | --- |
| ClinicalTrials.gov Identifier: | [NCT00872417](http://clinicaltrials.gov/ct2/show/NCT00872417) [History of Changes](http://www.clinicaltrials.gov/ct2/archive/NCT00872417) |
| Other Study ID Numbers: | CACT0810, PUMCH |
| Study First Received: | March 30, 2009 |
| Last Updated: | March 30, 2009 |
| Health Authority: | United States: Food and Drug Administration |

Keywords provided by Peking Union Medical College:

| HIV/AIDS antiretroviral therapy immune reconstitution |
| --- |

Additional relevant MeSH terms:

| HIV Infections Acquired Immunodeficiency Syndrome Anti-HIV Agents Lentivirus Infections Retroviridae Infections RNA Virus Infections Virus Diseases Sexually Transmitted Diseases, Viral Sexually Transmitted Diseases Immunologic Deficiency Syndromes Immune System Diseases | Slow Virus Diseases Lamivudine Reverse Transcriptase Inhibitors Nucleic Acid Synthesis Inhibitors Enzyme Inhibitors Molecular Mechanisms of Pharmacological Action Pharmacologic Actions Anti-Retroviral Agents Antiviral Agents Anti-Infective Agents Therapeutic Uses |
| --- | --- |

ClinicalTrials.gov processed this record on June 14, 2012
